# Clinical Characteristics and Outcomes of Neurosurgical Patients at a Level III Intensive Care Unit in Pakistan: A Retrospective Cohort Study

**DOI:** 10.7759/cureus.52990

**Published:** 2024-01-26

**Authors:** Atqua Sultan, Muhammad F Khan, Muhammad Sohaib, Faisal Shamim

**Affiliations:** 1 Anesthesiology, Nishtar Medical University/Hospital Multan, Multan, PAK; 2 Anesthesiology, The Aga Khan University, Karachi, PAK; 3 Anesthesiology, The Aga Khan University Hospital, Karachi, PAK

**Keywords:** apache-ii, intensive care, mechanical ventilation, outcomes, neurocritical care

## Abstract

Objective

Neurosurgical patients account for the majority of cases across all surgical specialties that are admitted to the surgical intensive care unit (ICU) at our institution. The goal of this study was to analyze factors leading to ICU admission, type of neurosurgical intervention, length of ICU/hospital stays, and outcomes in terms of complications and ICU and in-hospital mortality.

Methods

This retrospective study conducted at the surgical ICU, Aga Khan University Hospital, investigated clinical data of neurosurgical patients admitted between January 2020 and June 2022. Quantitative data were collected regarding patients’ characteristics, such as age, gender, comorbidities, type of surgical intervention, mode of surgery, source of admission to ICU, and type of osmotherapy. The primary and secondary outcomes were in terms of ICU and hospital mortality and complications.

Results

Among 321 patients admitted to the SICU, 197 were included according to inclusion/exclusion criteria. A total of 168 patients (85.3%) required surgical intervention, of whom 101 (60%) underwent elective surgery and 67 (40%) required emergency surgery. Thirteen patients died during the ICU or hospital stay, representing a mortality rate of 6.6%. The average length of stay in the ICU had a median IQR of 4 (4,6) days while the average hospital stay median IQR was 11 (12,18) days. Tracheostomy was performed in 77 patients (39%), and the median IQR day for tracheostomy was 4 (3,5) days. APACHE-II (Acute Physiology and Chronic Health Evaluation) score, length of ICU, and length of hospital stay were significantly higher in the deceased patients with a p-value of 0.042, 0.019, and 0.043, respectively.

Conclusion

In conclusion, this study on neurosurgical patients from the surgical intensive care unit of a low-middle-income country provided valuable insights about factors and their influence on outcomes. The study implies that a high APACHE-II score is linked to poorer outcomes for neurosurgical patients in this particular setting. Undertaking a large multicenter prospective study is vital for tailoring interventions and improving patient care in regions with limited resources where healthcare challenges may be distinct.

## Introduction

Neurosurgical disorders are the major cause of morbidity and mortality in the surgical intensive care unit (ICU). The majority of patients who are admitted to the ICU suffer from neurological disorders, including traumatic brain injuries (TBIs), spontaneous hematomas, ischemic strokes, and subarachnoid hemorrhages (SAH) [1]. TBIs are the most common type of brain injury worldwide. The prevalence of TBI ranges from 55 to 69 million but disproportionately affects people in low-middle-income countries due to limited resources and a speculative health burden [2].

The reason for the improved outcomes of neurosurgical patients is because of the development of different trauma centers having advanced modalities for managing such patients. The modalities are of medical, radiological, and surgical support and services, i.e. intracranial pressure monitoring possibilities, craniotomy expertise, etc., in the case of TBI [3]. Furthermore, the ratio of the number of patients treated to ICU beds, the ratio of ICU beds to hospital beds, and the nurse-to-patient ratio are all characteristics that have been proven to affect outcomes in mixed cohorts of ICU patients [4].

The fact is that the admission of patients to the ICU from the operating room (OR) or emergency room (ER) is challenging, as the availability of beds is the utmost important factor. In the ICU, the provision of extensive diagnostics and mechanical ventilation, including weaning protocols, are the prime reasons often observed for the prolonged ICU stay and the high cost of care. The care is impacted by the presence of an intensivist, critical care physician, or even a neurocritical care physician.

From January 2009 to June 2009, a retrospective audit at Agha Khan University Hospital revealed a significant death rate owing to moderate to severe TBI, which could be lowered by establishing evidence-based guidelines for pre-hospital and in-hospital interventions [5]. Another retrospective study was done in the Pediatric ICU of Agha Khan University Hospital, which assessed the burden and spectrum of neurological illness and concluded that central nervous system infections, status epilepticus, and severe traumatic brain injuries were the most common acute neurological illnesses in their cohort and were associated with higher mortality [6].

Our study aimed to review the outcomes of all neurosurgical patients admitted to the general surgical intensive care unit. We compared the study results with evidence in the literature to develop strategies for improving the care of this special cohort of patients in the general surgical intensive care unit. The primary objective of the study was to determine the ICU and hospital mortality rate of all neurosurgical patients in general surgical intensive care and the secondary outcome was to determine the length of ICU and hospital stay, ICU-acquired infections, and tracheostomy in the ICU along with other parameters.

## Materials and methods

**Figure 1:**
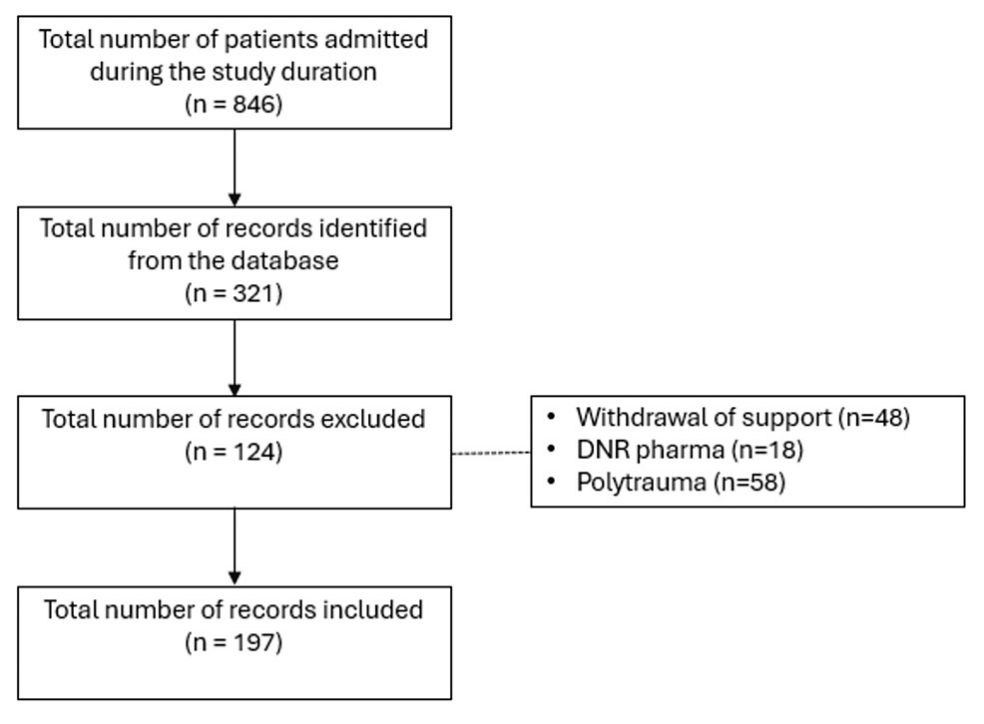
Flow chart for the number of included records DNR: do not resuscitate

For this retrospective observational cohort study, the sample size included all neurosurgical patients admitted to the surgical ICU during the study period, i.e., January 2020 - June 2022. The total number of patients admitted to the Surgical ICU was 846 and the total number of records identified from the database was 321 patients. It makes up 37.94% of Neurosurgical Patients’ admission to the Surgical ICU. 124 patients were excluded because they didn’t meet the inclusion criteria as shown in the flow chart (Figure 1).

Data were collected for all patients who met the inclusion criteria. The exclusion criteria consisted of the patients who had polytrauma, who died in <24 hours, and who were considered for do not resuscitate (DNR)/withdrawal of care. After approval was taken from the Ethical Review Committee of The Agha Khan University Hospital (2022-8024-22969), the primary investigator collected the data. All information was obtained from the patient’s medical record as per the designed proforma, which comprised demographics, mode of surgery (elective vs emergency), source of admission to ICU, osmotherapy, and type of intervention. The data collection of primary and secondary outcomes looked for mortality, the requirement of vasopressors/inotropes, hospital and ICU stay, ICU-acquired infections, acute kidney injury (AKI), arrhythmias, and tracheostomy. Besides, the APACHE-II score was also calculated.

Statistical analysis

Data were analyzed in R-Studio 4.1.2. (R-Foundation for Statistical Computing, Vienna, Austria). Based on an investigation into the nature of the normality assumption, mean/median, and standard deviation/IQR (inter-quartile range) were computed for the quantitative characteristics of patients, including age, length of ICU, and hospital stay. Gender, comorbidities, patients requiring inotropes/vasopressors, ICU mortality, infections acquired during ICU stay, tracheostomy in the ICU, and hospital mortality were computed for frequency and percentage for qualitative characteristics.

The assumption of normality for the numerical variables was tested using the Shapiro-Wilk or Kolmogorov-Smirnov test. To determine the effect of ICU care for neurosurgical patients on mortality rate, the patients were categorized into two groups, namely, survived and deceased, followed by their comparison. The independent t-test/Mann-Whitney test was applied depending on whether the variable is normal or non-normal while the chi-square/Fisher's exact test was used to compare the categorical variables. A p-value of less than 0.05 was considered significant.

## Results

The results of our study showed that out of 197 patients, 168 (85.3%) underwent surgical intervention and the types and modes of surgical intervention are shown in Table 1, along with the demographics and other clinical characteristics.

**Table 1:** Demographics and clinical characteristics of neurosurgical patients in the ICU ⸸Mean ± standard deviation: quantitative variable; *n (%): qualitative variable

General surgical ICU	Categories	Frequency (%)
Source of admission to ICU^*^	Operating room	164 (82.8%)
	Emergency room	26 (13.1%)
	Wards/special care	6 (3%)
Patient demographics and characteristics		
Age (years)^⸸^	Mean ± S.D	42.8 ± 15.7
	Median (IQR)	42 (30, 55)
Gender^*^	Male	150 (76%)
Comorbidities^*^	None	105 (53%)
	Hypertension	74 (37.4%)
	Diabetes mellitus	38 (19.2%)
	Ischemic heart disease	16 (8.1%)
	Chronic kidney disease	2 (1%)
	Chronic liver disease	1 (0.5%)
	Other	23 (11.6%)
Number of cases^*^	Traumatic brain injury	96 (48.7%)
	Non-traumatic brain injury	101 (51.3%)
Type of management^*^	Surgical intervention	168 (85.3%)
	Conservative management	29 (14.7%)
Mode of surgery^*^ (n=168)	Elective	101 (60%)
	Emergency	67 (40%)
Type of surgical intervention^*^ (n=168)	Craniotomy	88 (52%)
	Craniectomy	36 (21.4%)
	Other	44 (26.2%)
Osmotherapy^*^	Yes	44 (22.2%)
Type of osmotherapy^*^ (n=44)	Mannitol	3 (6.8%)
	3% hypertonic saline	28 (63.6%)
	Combined	13 (29.5%)

The primary and secondary outcomes are shown in Table 2. The total mortality was 13 (6.6%) patients and the APACHE-II (Acute Physiology and Chronic Health Evaluation) score median IQR was 13 (11,16). The median IQR for the ICU and hospital stay was 4 (4,6) and 11 (12,18) days, respectively. Tracheostomy was carried out in 77 (39%) patients and the median (IQR) for the day of tracheostomy was 4 (3, 5). Besides this, 4 (2%) patients had developed new-onset arrhythmias during the ICU stay. At least, 21 (10.6 %) required vasopressors/inotropes to maintain the hemodynamics, and 14 (7.1%) patients had developed AKI.

**Table 2:** Outcomes of neurosurgical patients in the ICU APACHE-II: Acute Physiology and Chronic Health Evaluation

Outcomes		Frequency(%)
ICU mortality^*^	NA	1 (0.5%)
Hospital mortality^*^	NA	12 (6.1%)
Total mortality cases^*^	NA	13 (6.6%)
APACHE-II score^⸸^	Mean ± S.D	12.5 ± 2.6
	Median (IQR)	13 (11, 16)
Length of ICU stay (days)^ ⸸^	Mean ± S.D	3.94 ± 1.6
	Median (IQR)	4 (4, 6)
Length of hospital stay (days)^ ⸸^	Mean ± S.D	11.58 ± 5.8
	Median (IQR)	11 (12, 18)
New onset arrhythmias^*^	NA	4 (2%)
New onset acute kidney injury^*^	NA	14 (7.1%)
Patients requiring inotropes/vasopressors during ICU stay^*^	NA	21 (10.6%)
Tracheostomy^*^	NA	77 (39%)
Day of tracheostomy^⸸^	Mean ± S. D	4 ± 1
	Median (IQR)	4 (3, 5)

**Figure 2:**
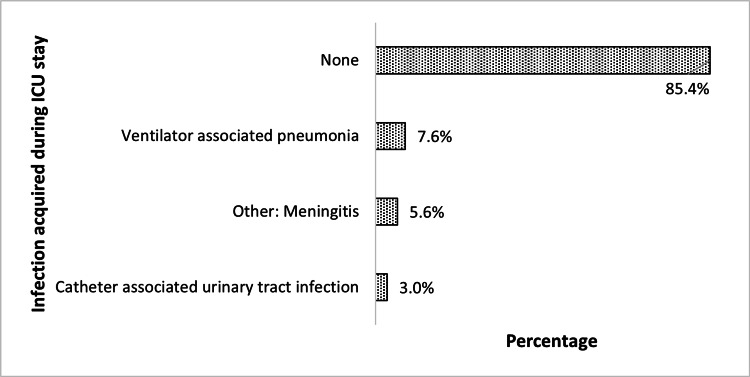
Percentage of infections acquired during the ICU stay

The infections acquired during the ICU stay are shown in Figure 2. The highest percentage is for ventilator-associated pneumonia followed by meningitis and catheter-associated urinary tract infections.

A comparison of deceased and alive patients is shown in Table 3. The patients who died during the ICU or hospital stay had more comorbidities, a higher APACHE-II score, and prolonged ICU and hospital stays. The p-value for APACHE-II was 0.042 and for ICU and hospital length of stay, it was 0.019 and 0.043, respectively, and was significant.

**Table 3:** Comparison of deceased and alive patients APACHE-II: Acute Physiology and Chronic Health Evaluation

Variables		Deceased	Alive	p-value
Gender	Male (n=150)	11 (7.3%)	139 (92.7%)	0.737
	Female (n=47)	2 (4.3%)	45 (95.7%)	
Comorbidities	Hypertension (n=74)	8 (10.8%)	66 (89.2%)	0.079
	Diabetes (n=38)	6 (15.8%)	32 (84.2%)	0.021
	Ischemic heart disease (n=16)	3 (18.8%)	13 (81.3%)	0.076
Mode of surgery (n=168)	Elective (n=101)	5 (5%)	96 (95%)	0.349
	Emergency (n=67)	6 (9%)	61 (91%)	
APACHE-II score	Mean rank	129.62	96.84	0.042
LOS-ICU (days)	Mean rank	134.15	96.52	0.019
LOS-hospital (days)	Mean rank	129.81	96.82	0.043

## Discussion

The current study looked at the outcomes of neurosurgical patients in Agha Khan University Hospital’s surgical intensive care unit. In our study, the male population outnumbered the female in a ratio of 3*to1*and the median (IQR) of the age group was 42 (30,55). Most patients were admitted for post-operative care after emergency and elective neurosurgeries. In a retrospective study by Babatunde et al., neurosurgical admissions made up a significant percentage of ICU admissions, i.e. 33.2%, with traumatic brain injury and post-craniotomy for tumor excision being predominant indications [7].

Approximately 47% (92) of our study population was suffering from co-morbidity. It can be challenging to gather a thorough history of a trauma patient's underlying comorbid diseases; in many circumstances, the treating physician's only resources are test results and a clinical examination. In patients with traumatic epidural hematoma, several comorbidities were found to be associated with various outcome indicators [8]. This makes intuitive sense because patients with additional comorbidities are less healthy than patients with simply the core condition for which they are being treated, and comorbidities increase the likelihood of complications, both medical and surgical. In our study, we found a similar linear association between outcomes and comorbidity, with more co-morbidity being associated with mortality.

The number of cases of traumatic and other neurosurgical cases was 48.7% (96) and 51.3% (101), respectively. Subdural, epidural, and subarachnoid hemorrhages were the most frequent clinical findings. A study by Waqar et al. assessed the risks and benefits of the surgical treatment (open craniotomy) of intra-cerebral hematoma with all the patients being admitted to ICU for postoperative monitoring and showed that presenting GCS with early surgical evacuation carried out better results [9]. In our study, 168 (85.3%) patients underwent surgical intervention while 29 (14.7%) were managed conservatively. One-hundred sixty-four (164; 82.8%) patients were admitted to the ICU from the operating room after undergoing surgery, 101 (60%) underwent elective surgeries, and 67 (40%) underwent emergency surgeries, respectively.

According to a study by Siqueira et al., the total mortality rate after elective neurosurgery was reported to be 1%, compared to 29% after emergency neurosurgery, with postoperative complications increasing the risk of death in both groups [10]. Although there is little research on length-of-stay factors, especially for TBI patients, prior studies have found that age, gender, Glasgow Coma Scale, injury severity, and mechanical ventilation are significant risk factors for prolonged LOS [11]. The average length of ICU and hospital stay in our study had a median IQR of 4 (4,6) and 11 (12,18), respectively.

The study by Lazaridis et al. concluded that intracranial pressure (ICP), as measured and controlled by Brain Trauma Foundation recommendations, had no relationship with ICU duration of stay. Regardless of injury severity or intracranial pressure course, patients with severe traumatic brain injury who had a mass lesion on admission head computed tomography had a lengthier ICU stay [12].

According to Knaus et al., only 15% of the neurosurgery patients brought to an ICU postoperatively received active treatment [13]. In our study, the median IQR of the APACHE-II score calculated was 13 (11,16) indicating that the risk of death is greater than 15%. However, our findings revealed a 6.6% mortality rate in our study. This is like the findings of Phuping et al., who discovered that not only did the APACHE-II score in neurosurgical patients indicate low severity, but it also performed poorly in predicting hospital mortality. More research into predicting mortality in these critical patients is needed [14].

For the medical management of intracranial pressure (ICP), 22.2% (44) of patients received hyperosmolar therapy. Rickard and colleagues performed a meta-analysis to compare the use of mannitol and hypertonic saline (HTS) in hemodynamically stable adults with traumatic brain injury. The authors discovered a trend towards better intracranial pressure control with hypertonic saline but concluded that both mannitol and hypertonic saline are equally effective at reducing increased intracranial pressure, with no statistically significant difference in overall performance [15].

The average day on which tracheostomy was performed in ICU patients had a median IQR of 4 (3,5). The best time to perform a tracheotomy to deliver favorable clinical results for critically ill patients who need mechanical ventilation is still up for debate despite multiple clinical research. According to the mentioned study, early tracheostomy was linked to decreased mechanical ventilation (MV) days and ICU length of stay, as well as a lower incidence of ventilator-associated pneumonia (VAP), in critically ill neurosurgical patients [16].

Patients in the intensive care unit (ICU) are at risk of healthcare-associated infections (HAIs), particularly surgical site infections (SSIs) and other types of treatment-associated infections. Infection rates in our study sample were 7.6% (15) for ventilator-associated pneumonia development, 5.6% (11) for meningitis, and 3% (6) for catheter-associated urinary tract infection (CAUTI), respectively. The risk factors for hospital-acquired infections include the insertion of central venous catheters, urinary catheters, and mechanical ventilation. In comparison to other research conducted in other countries, the surveillance study in a Neuro Critical Care Unit (NCCU) of a developing country hospital revealed comparable rates of overall ICU-acquired infections in our patient population (9.6 compared with 3.7-25.0 per 100 patients) [17].

Arrhythmias appears to be a rare diagnosis for primary ICU admission. Two percent (2%; 4) of patients in our study developed new-onset arrhythmia during the ICU stay. Arrhythmias existence will depend on the case mix and any underlying diseases. In a surgical ICU, the annual incidence was 14.9%, and in a medical ICU, it ranged from 15.7% to 19.7%, according to prospective observational studies [18]. AKI developed in 7.1% (14) of neurosurgical patients in our ICU. AKI was found in 23.5% of neurocritical patients in a retrospective study by Guerrero et al. [19].

Critically ill patients routinely get inotropes and vasopressors to treat hemodynamic abnormalities and ensure appropriate organ perfusion. Our results showed that 10.6% (21) of patients required inotropes and vasopressors during the ICU stay. According to Justina et al., patients who received vasoactive medicines in the beginning days of their ICU stay had a greater chance of mortality in the ICU. This is consistent with the results of our study that out of 13 patients who died five patients required inotropes and vasopressors at some point during their stay [20].

Limitations

First, it’s a single-center retrospective study. Second, due to our study focus, we didn’t include the data for patients who underwent polytrauma, withdrawal of care, and no escalation of treatment at some stage during the ICU admission. Also, we didn’t monitor the ICP of the patients and didn’t look for the outcome of the patients at six months. We didn’t count the patients who were shifted from ICU on portable ventilators and discharged to home on the ventilator.

## Conclusions

In conclusion, this study on neurosurgical patients from the surgical intensive care unit of a low-middle-income country provided valuable insights about factors and their influence on outcomes. The study implies that a high APACHE-II score is linked to poorer outcomes for neurosurgical patients in this particular setting. Undertaking a large multicenter prospective study is vital for tailoring interventions and improving patient care in regions with limited resources where healthcare challenges may be distinct.
